# Cerebral Amyloidosis in Individuals with Subjective Cognitive Decline: From Genetic Predisposition to Actual Cerebrospinal Fluid Measurements

**DOI:** 10.3390/biomedicines12051053

**Published:** 2024-05-10

**Authors:** Stefanos N. Sampatakakis, Niki Mourtzi, Sokratis Charisis, Faidra Kalligerou, Eirini Mamalaki, Eva Ntanasi, Alex Hatzimanolis, Georgios Koutsis, Alfredo Ramirez, Jean-Charles Lambert, Mary Yannakoulia, Mary H. Kosmidis, Efthimios Dardiotis, Georgios Hadjigeorgiou, Paraskevi Sakka, Konstantinos Rouskas, Kostas Patas, Nikolaos Scarmeas

**Affiliations:** 1 1st Department of Neurology, Aiginition Hospital, Athens Medical School, National and Kapodistrian University, 11528 Athens, Greece; stefanos.sab@gmail.com (S.N.S.); nikimourtzi23@gmail.com (N.M.); fkalliger@gmail.com (F.K.); eir.mamalaki@gmail.com (E.M.); e.ntanasi@hotmail.com (E.N.); 2 Department of Neurology, UT Health San Antonio, San Antonio, TX 78229, USA; scharissis@gmail.com; 3 Department of Psychiatry, Aiginition Hospital, Athens Medical School, National and Kapodistrian University, 11528 Athens, Greece; alhatzi@gmail.com; 4 Neurogenetics Unit, 1st Department of Neurology, Aiginition Hospital, Athens Medical School, National and Kapodistrian University, 11528 Athens, Greece; gkoutsis@med.uoa.gr; 5 Division of Neurogenetics and Molecular Psychiatry, Department of Psychiatry and Psychotherapy, Medical Faculty, University of Cologne, 50923 Cologne, Germany; alfredo.ramirez@uk-koeln.de; 6 Department of Neurodegenerative Diseases and Geriatric Psychiatry, University Hospital Bonn, 53127 Bonn, Germany; 7 German Center for Neurodegenerative Diseases (DZNE Bonn), 53127 Bonn, Germany; 8 Department of Psychiatry, Glenn Biggs Institute for Alzheimer’s and Neurodegenerative Diseases, San Antonio, TX 78229, USA; 9 Excellence Cluster on Cellular Stress Responses in Aging-Associated Diseases (CECAD), University of Cologne, 50923 Cologne, Germany; 10 Inserm, CHU Lille, Institut Pasteur de Lille, U1167-RID-AGE Facteurs de Risque et Déterminants Moléculaires des Maladies Liés au Vieillissement, University of Lille, 59000 Lille, France; jean-charles.lambert@pasteur-lille.fr; 11 Department of Nutrition and Dietetics, Harokopio University, 17676 Athens, Greece; myianna@hua.gr; 12 Lab of Neuropsychology and Behavioral Neuroscience, School of Psychology, Aristotle University of Thessaloniki, 54124 Thessaloniki, Greece; kosmidis@psy.auth.gr; 13 Department of Neurology, University Hospital of Larissa, Faculty of Medicine, School of Health Sciences, University of Thessaly, 41334 Larissa, Greece; edar@med.uth.gr; 14 Department of Neurology, Medical School, University of Cyprus, Nicosia 1678, Cyprus; gmhadji@med.uth.gr; 15 Athens Association of Alzheimer’s Disease and Related Disorders, 11636 Marousi, Greece; vsakka@ath.forthnet.gr; 16 Institute of Applied Biosciences, Centre for Research & Technology Hellas, 54124 Thessaloniki, Greece; rouskas@certh.gr; 17 Department of Medical Biopathology and Clinical Microbiology, Aiginition Hospital, Athens Medical School, National and Kapodistrian University, 11528 Athens, Greece; konpatas@gmail.com; 18 Department of Neurology, The Gertrude H. Sergievsky Center, Taub Institute for Research in Alzheimer’s Disease and the Aging Brain, Columbia University, New York, NY 10027, USA

**Keywords:** subjective cognitive decline, genetics, Alzheimer’s disease, biomarkers

## Abstract

The possible relationship between Subjective Cognitive Decline (SCD) and dementia needs further investigation. In the present study, we explored the association between specific biomarkers of Alzheimer’s Disease (AD), amyloid-beta 42 (Aβ_42_) and Tau with the odds of SCD using data from two ongoing studies. In total, 849 cognitively normal (CN) individuals were included in our analyses. Among the participants, 107 had available results regarding cerebrospinal fluid (CSF) Aβ_42_ and Tau, while 742 had available genetic data to construct polygenic risk scores (PRSs) reflecting their genetic predisposition for CSF Aβ_42_ and plasma total Tau levels. The associations between AD biomarkers and SCD were tested using logistic regression models adjusted for possible confounders such as age, sex, education, depression, and baseline cognitive test scores. Abnormal values of CSF Aβ_42_ were related to 2.5-fold higher odds of SCD, while higher polygenic loading for Aβ_42_ was associated with 1.6-fold higher odds of SCD. CSF Tau, as well as polygenic loading for total Tau, were not associated with SCD. Thus, only cerebral amyloidosis appears to be related to SCD status, either in the form of polygenic risk or actual CSF measurements. The temporal sequence of amyloidosis being followed by tauopathy may partially explain our findings.

## 1. Introduction

Recently, complaints regarding cognitive function have shown an increasing tendency in the general population leading to the conception of Subjective Cognitive Decline (SCD) [1], a medical term used for the description of self-reported experience of cognitive worsening. Throughout the last decade, many research efforts have focused on describing SCD types and characteristics; nevertheless, to date, no clinical instrument is able to differentiate individuals with and without SCD. More specifically, there are no widely accepted scales or questionnaires to estimate SCD, as existing questionnaires present problems of internal consistency and content validity [2]. Consequently, SCD has not yet been recognized as a medical entity in the Diagnostic and Statistical Manual of Mental Disorders [3]. SCD is considered an early pre-dementia stage [4], as existing evidence suggests that SCD might precede objective memory decline. In fact, self-reported or partner-reported subjective memory complaints (SMCs) have been associated with an increased risk of cognitive decline [5], while self-reported subjective cognitive complaints (SCCs) have also been related to increased rates of Alzheimer’s and non-Alzheimer’s disease (AD) dementia [6].

Abnormal values of cerebrospinal fluid (CSF) biomarkers of Alzheimer’s disease (AD) have appeared to precede the development of objective cognitive decline [7]. This underscores why CSF biomarkers might be considered sensitive indicators of underlying pathophysiology in preclinical dementia stages [8] and probably useful in determining whether SCD is indeed linked to AD-related pathological changes. More specifically, CSF amyloid-beta 42 (Aβ_42_) pathology in SCD individuals has been consistently associated with worsening cognitive decline [9] and clinical progression to dementia [10], with Aβ_42_ being one of the strongest predictors of clinical conversion in individuals with SCD [11]. Moreover, SCD individuals with amyloid-positive profiles according to the ATN classification have been related to higher dementia risk in comparison to amyloid-negative profiles [12]. Regarding Tau, only one study found a significant association between CSF Tau levels (as an independent predictor) and the development of cognitive impairment [13]. 

Taking all the above into consideration, the existing literature has provided clear conclusions regarding the association between CSF biomarkers and clinical progression in individuals with SCD, as those with amyloid pathology are more likely to develop objective cognitive decline longitudinally. However, it remains unknown whether people who complain about their memory or cognition have abnormal measurements of AD biomarkers at the moment of complaining (cross-sectionally) and, thus, belong in the AD biological continuum. Associations between SCD status and low CSF Aβ_42_ have been reported [14], as well as high Aβ_42_ levels in positron emission tomography (PET), either as a continuous [15] or a categorical variable, as depicted by amyloid positivity [16]. However, cross-sectional data relating Aβ_42_ and Tau with the odds of SCD prevalence are limited. We are aware of no study associating CSF biomarkers of neurodegeneration, such as Tau, with SCD. Additionally, studies relating SCD with a genetic predisposition for Aβ_42_ or tauopathy have not been conducted. 

Therefore, in the current study, we sought to investigate whether the values of AD biomarkers are abnormal in individuals complaining about their memory or cognitive status. To achieve this aim, we explored whether AD biomarkers (i.e., Aβ_42_ and Tau) may affect the odds of SCD, combining data from two ongoing studies: the Hellenic Longitudinal Investigation of Aging and Diet (HELIAD) and the Aiginition Longitudinal Biomarker Investigation of Neurodegeneration (ALBION). The HELIAD study is population-based, including individuals οver 64 years old, in which genetic propensity for AD biomarkers is evaluated through relevant polygenic risk scores (PRSs). The ALBION study includes individuals 40–75 years old worrying about their cognitive status in whom CSF biomarker measurements are obtained. To our knowledge, our study is the first to examine the association of AD risk factors (Aβ_42_ and Tau) with SCD using two proxies, including CSF measurements as well as common variant polygenic risk estimation.

## 2. Materials and Methods

### 2.1. Participants and Study Design

Data for the analyses were drawn from the HELIAD and the ALBION, two ongoing studies examining cognitive disorders. Study procedures were approved by the Institutional Ethics Review Boards of the National and Kapodistrian University of Athens and the University of Thessaly. 

HELIAD participants were individuals older than 64 years old, randomly recruited from local municipality registries of Marousi, an Athens suburb, and the city of Larissa and rural surroundings. Overall, 1986 individuals completed the baseline evaluation of HELIAD. In the present analyses, we only included participants with available genetic data through blood sampling (*n* = 1189). Participants with a medical history of neurological disorders with a risk of cognitive impairment (cerebrovascular disease and stroke, severe traumatic brain injury, hydrocephalus, epilepsy, extrapyramidal disorders such as Parkinson’s disease, Huntington’s disease), psychiatric disorder (psychosis, major depression, or anxiety), alcoholism or drug abuse were not included in our analyses (*n* = 239), as well as participants with MCI or dementia at baseline (*n* = 208). Moderate depression, which is common in old age, as well as the use of antiepileptic or anxiolytic drugs at a steady dosage for years, were allowed. In total, 742 cognitively normal (CN) individuals were included. Extensive details about the design and key features of the HELIAD study design and data collection procedure have been described previously [17].

The ALBION study sample consisted of individuals aged >40 years old, referred to the cognitive disorders’ outpatient clinic of Aiginition Hospital (Athens, Greece). Patients with a dementia diagnosis were excluded from ALBION, as well as patients with medical conditions associated with a high risk of cognitive impairment or dementia (including Parkinson’s disease, severe stroke, multiple sclerosis, hydrocephalus, epilepsy, Huntington’s disease, Down syndrome, active alcohol or drug abuse or major psychiatric conditions such as major depressive disorder, schizophrenia, and bipolar disorder). The baseline evaluation was completed by 198 individuals from 2019 to 2023. In the present analyses, we only included ALBION participants in their baseline evaluation with available CSF biomarker results (*n* = 185). Among these, 13 participants were excluded due to prior history of stroke or severe traumatic brain injury, while 65 individuals were excluded due to Mild Cognitive Impairment (MCI). Overall, 107 cognitively normal (CN) individuals were included in our study. More details about the study design and data collection procedures can be found elsewhere [18].

### 2.2. Neurological and Neuropsychological Evaluation

HELIAD and ALBION participants underwent a comprehensive neuropsychological evaluation during their baseline assessment, conducted by licensed neuropsychologists. The following tests were used to assess participants’ cognition: the Mini Mental State Examination [19], the Greek Verbal Learning Test [20], the Medical College of Georgia Complex Figure Test (copy condition, recognition, immediate and delayed recall and recognition) [21], a semantic and phonological verbal fluency test [22], subtests of the Greek version of the Boston Diagnostic Aphasia Examination short form and selected items from the Complex Ideational Material Subtest [23], the Greek Trail Making Test [24], an abbreviated form of Benton’s Judgment of Line Orientation [25], and the Clock Drawing Test [26], as well as a graphical sequence task and motor programming [27]. 

Then, we constructed a composite neuropsychological score based on the results of all the aforementioned test in order to assess global cognition. A higher neuropsychological score indicates a better cognitive performance. The specific score, which is a non-weighted average of the individual tests, was used as a covariate in the statistical analyses. 

A comprehensive neurological examination was performed by certified neurologists, while participants were also asked to provide information concerning medical and family history and lifestyle as well as demographic data during the baseline evaluation. Finally, neurologists established a clinical diagnosis, taking all the above-mentioned information into consideration. In fact, MCI diagnosis in both studies was based on standard international criteria (Petersen criteria [28], i.e., presence of SMC as well as objective impairment in at least one cognitive domain with preserved activities of daily living and absence of dementia), while the NINCDS/ADRDA criteria [29] were used to establish an AD diagnosis.

### 2.3. Subjective Cognitive Decline (SCD) Assessment

In the absence of widely accepted scales or questionnaires to assess SCD [30], we combined information from a series of single questions derived from relevant questionnaires such as the Blessed Dementia Scale, the Lawton IADL scale, and the CARE Subjective Memory subscale and then grouped similar questions together. Answers were obtained based on the self-reporting of relevant complaints. 

We created dichotomous variables reflecting Subjective Cognitive Decline (SCD) based on the participant’s response to two questions assessing SCC. Each question had a possible rating of (0) “I don’t have this complaint” or (1) “I have this complaint”, while a positive answer to at least one of them resulted in a score of 1 (dichotomous variable). The questions used in each study are presented in detail in Table 1. 

### 2.4. Cerebrospinal Fluid (CSF) Analysis in ALBION 

CSF was collected and stored according to widely accepted guidelines [31], while each participant underwent a lumbar puncture in the context of the first ALBION evaluation. The collected CSF samples were analyzed using automated Elecsys assays (Roche Diagnostics), mainly for AD biomarkers such as amyloid Aβ_42_, Tau, and P-Tau. A positive result was noted according to the following reference ranges: Aβ_42_ ≤ 1000 pg/mL, Tau > 300 pg/mL, and P-Tau > 27 pg/mL.

### 2.5. Genotyping and Imputation in HELIAD

Genome-wide genotyping in HELIAD was performed at three different centers (the Centre National de Recherche en Génomique Humaine [CNRGH, Evry, France] and the Life and Brain Center [Bonn, Germany], as well as the Erasmus Medical University [Rotterdam, The Netherlands]) using the Illumina Infinium Global Screening Array, as part of the European Alzheimer & Dementia Biobank (EADB) project. Genotyping in HELIAD has been described in detail elsewhere [32]. More information is provided in the Supplementary Materials. 

### 2.6. Polygenic Risk Score (PRS) Calculation in HELIAD 

Genetic predisposition for CSF Aβ_42_ and plasma total Tau levels were based on two specific PRSs that were formed by aggregating the effect of common variants associated with Aβ_42_ and Tau levels, respectively. The PRS Aβ_42_ was constructed based on the summary statistics results of a genome-wide association study (GWAS) for CSF Aβ_42_ [33], while the PRS Tau was calculated based on summary statistics results of a GWAS meta-analysis for circulating total Tau levels [34]. 

In the GWAS summary statistics, each SNP is associated with CSF Aβ_42_ or total Tau levels at a certain *p*-value threshold. For each participant, we computed different PRSs based on a prior set of 10 *p*-value GWAS thresholds (P_T_) (i.e., 5 × 10^−5^, 0.0001, 0.001, 0.05, 0.01, 0.1, 0.2, 0.3, 0.4, 0.5) for Aβ_42_ and 11 P_T_ for total Tau (the aforementioned thresholds plus the 5 × 10^−8^ threshold). The numbers of SNPs at each GWAS P_T_ included for PRSs calculation are provided in Tables S1 and S2, while additional information regarding the methods of calculation can be found in the Supplementary Materials. Given that Aβ_42_ levels in CSF are inversely related to brain Aβ_42_ concentration, PRS values were multiplied by –1 to align a higher score with increased Aβ_42_ levels in the brain.

### 2.7. Statistical Analysis

The statistical analyses were performed using SPSS 29.0. Participants’ characteristics were expressed as mean values ± standard deviation (SD) for continuous variables or as percentages for categorical variables. Groups according to presence of SCD (SCD and non-SCD group) were compared using analysis of variance (ANOVA) for continuous variables such as age, education years, and neuropsychological score, and Fisher’s exact test for categorical variables such as sex and depression. The significance level was set at *p* < 0.05. 

For our analyses, we selected the PRS thresholds with the best classification accuracy of amnestic MCI (aMCI) or AD versus non-aMCI or AD cases, assuming that individuals with aMCI or AD at follow-up were the most likely to have pathologic levels of CSF Aβ_42_ as well as pathologic plasma total Tau levels. For this specific purpose, logistic regression models were used with the different P_T_s as predictors and incident aMCI or AD at follow-up as outcome. The first two principal components (PC1, PC2) of genetic ancestry were used as covariates in order to eliminate the possibility of cryptic relatedness between participants [35] or unexpected errors related to genotyping batch [36]. P_Ts_, which were better able to discriminate between the presence versus absence of Aβ_42_ and Tau pathology, were chosen based on the area under the receiver operator characteristic (ROC) curves constructed for each model (i.e., P_T_ < 0.0001 for Aβ_42_ consisting of 57 SNPs and P_T_ < 5 × 10^−8^ for Tau consisting of 21 SNPs). The results for all the thresholds analyzed can be found in Tables S1 and S2. 

CSF biomarkers were treated as dichotomous variables according to the above-mentioned Elecsys assay cut-offs (0: negative/normal result and 1: positive/abnormal result). PRSs were also treated as dichotomous variables, with the median used as a cut-off (0: low PRS and 1: high PRS). The values of the medians were 0.025 for PRS Aβ_42_ and −0.017 for PRS Tau, respectively. 

The association of CSF biomarkers and the PRSs with SCD was investigated with logistic regression analyses. SCD was selected as the outcome variable (No-SCD: 0 and SCD: 1). CSF measurements as well as PRSs were introduced into the models as dichotomous predictor variables (normal as reference for CSF and low as reference for PRSs, respectively). Our logistic regression analyses were adjusted for possible confounding factors such as age, sex (male vs. female), years of education, neuropsychological score, and depression (presence vs. absence), which were also inserted as predictors in our models. 

## 3. Results

### 3.1. Descriptive Statistics and Participants’ Demographics

The baseline demographic, clinical, and genetic characteristics of HELIAD participants are presented in Table 2. In HELIAD, 186 participants had SCD at baseline (25.1%). Individuals with SCD had more education years and lower neuropsychological scores in comparison to individuals not complaining about their memory (*p* < 0.001). Moreover, a higher percentage of SCD individuals had high PRS Aβ_42_ (*p* = 0.028) as well as depression (*p* = 0.030). The prevalence of high PRS Tau did not differ between the two groups. 

The baseline demographic, neuropsychological, and clinical characteristics of ALBION participants are presented in Table 3. In ALBION, 78 participants had SCD at baseline (72.9%). ALBION participants with SCD at baseline were more likely to have abnormal CSF Aβ_42_ values (*p* = 0.038) compared to those belonging in the non-SCD group. Other participant characteristics (including CSF Tau and CSF P-Tau) did not significantly differ between the two groups.

### 3.2. Factors Associated with the Odds of Prevalent SCD 

**Figure 1:**
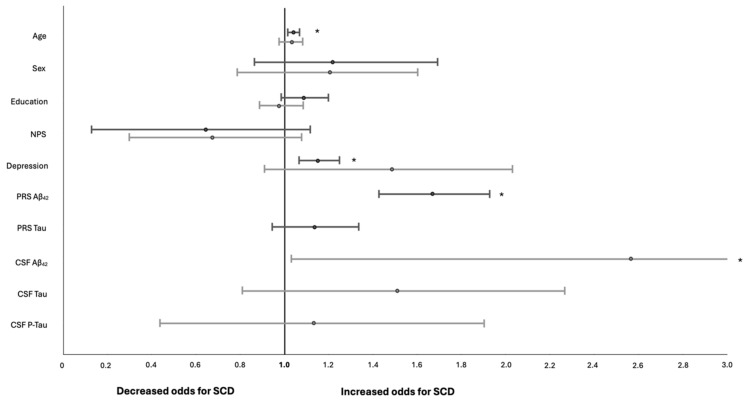
Odds Ratio Plots for SCD in HELIAD (upper plots) and ALBION (lower plots) with 95% confidence intervals (asterisks indicate statistically significant values).

High PRS Aβ_42_ increased the SCD odds up to 68.9% (*p* = 0.035) in HELIAD participants, while abnormal CSF Aβ_42_ values increased the odds for SCD by more than 2.5 times (*p* = 0.045) in ALBION participants. Neither high values of PRS Tau nor abnormal CSF Tau values were related to increased odds of SCD. Among other factors, age (2.7%, *p* < 0.001) and depression (13.3%, *p* = 0.027) increased the odds for SCD only in HELIAD participants. The results of the adjusted logistic regression models for the association of different factors with the odds of prevalent SCD in HELIAD and ALBION are shown in Table 4. ORs plots for SCD with 95% CIs in HELIAD and ALBION are shown in Figure 1.

## 4. Discussion

In our study, we found that, among CN individuals, abnormal values of CSF Aβ_42_ were related to 2.5-fold higher odds of SCD, while higher genetic predisposition to CSF Aβ_42_ was associated with 1.6-fold higher odds of SCD. Tau was not associated with the odds of SCD (neither actual CSF measurements nor total Tau PRS). Hence, the higher genetic risk for CSF Aβ_42_ deposition significantly increases SCD odds, a result which was independently validated in the context of real-time CSF measurements of Aβ_42_, with SCD odds being even higher.

The existing literature on the utility of AD biomarkers in the recognition of SCD as a preclinical stage of AD appears to be relatively limited. In particular, studies conducted in CN individuals have shown significant associations between SCD and lower CSF Aβ_42_ [17]. In these specific studies, neither CSF Tau nor CSF P-Tau levels were related to SCD. Lower CSF Aβ_42_ levels were also associated with cognitive decline in specific cognitive domains (i.e., memory and language) in the CN participants of the DELCODE study [37]. Furthermore, PET amyloid studies have related both amyloid-beta cortical binding as a continuous variable [15], as well as amyloid positivity as a categorical variable, with SMC [16]. Only one PET study [38], with a relatively small sample size (40 CN participants), has associated memory and organization decline with amyloid load in the frontal cortex, as well as decline in everyday planning with amyloid cortical binding in the parietal cortex.

Regarding Tau, the specific AD biomarker has been associated with SCD in only two studies. One of these showed statistically significant results, but included only individuals with preclinical autosomal dominant AD [39]; thus, the results have limited generalizability. Another PET study found that SCD was related to early tauopathy in the medial temporal lobe, specifically in the entorhinal cortex [40] of clinically healthy older adults. Nevertheless, the vast majority of studies conducted found no relationship. Apart from Aβ_42_ and Tau, several AD biomarker ratios have been associated with SCD, such as lower Aβ_42_/Tau ratio [38] and Aβ_42_/p-Τau ratio, both in CSF and PET measurements [41]. 

As far as the polygenic risk for AD biomarkers are concerned, relevant research regarding SCD is still in its infancy. To our knowledge, no study has examined the association between a PRS (either Aβ_42_ or Tau-specific) and SCD. Only a PRS including 39 genetic loci related to AD has been related to amyloid positivity in individuals with SCD [42]. 

Thus, our findings confirm the results of most studies regarding the association of CSF Aβ_42_ with SCD, as well as the absence of a relationship between Tau and SCD. Our results also expand existing knowledge by presenting the association of a PRS for Aβ_42_ and odds for SCD. These findings are in accordance with the ‘amyloid cascade’ hypothesis, which states that amyloid aggregation is the first event related to the neuropathology of AD [43] and, therefore, CSF Aβ_42_ becomes abnormal in the early stages with the patient still being CN without objective impairment. The accumulation of Tau might start emerging preclinically too, but later than amyloid [44]. Consequently, an increase in CSF Tau and total Tau plasma levels occurs in the later stages of the disease. A recent study concluded that CSF amyloid-beta (Aβ)_42_ preceded clinical diagnosis for 18 years, while total Tau preceded AD diagnosis for 10 years [45]. 

Taking the above into consideration, it appears that SCD might be an intermediate pre-clinical stage, between clinically normal older adults and MCI individuals, and possibly a pre-MCI condition [46]. A recent meta-analysis investigating the prevalence of amyloid pathology in older people on the AD continuum reported that amyloid pathology was more prevalent in SCD individuals (12%) than in CN individuals (10%) and less prevalent than in individuals with MCI (27%) and those aged 50 years [47]. Adding to this, our study suggests that, in this intermediate stage, AD-related pathophysiological changes might have already occurred (amyloid is already accumulated while Tau is not); however, these changes might not yet affect objective cognitive performance. Hence, SCD might reflect amyloid-induced sub-symptomatic decline, something that current neuropsychological test batteries are not sensitive enough to capture.

The present study has several strengths. To our knowledge, this is the first study to examine the link between SCD and genetic predisposition for Aβ_42_ and Tau, adding the PRS approach for AD biomarkers and combining it with actual CSF measurements in two different populations in order to replicate the observed results. Instead of relying solely on CSF results or a PRS, the results of our PRS approach were validated by actual assessments in clinical practice. Furthermore, well-established international criteria were used for clinical diagnosis, which was also supported by a comprehensive neuropsychological assessment. Finally, our analyses were adjusted for many potential confounders, which have been shown to be important risk factors for SCD [48], such as baseline global cognition as well as age, depression, and education.

Nevertheless, this specific study is not without limitations. Firstly, due to the inconsistency of SCD definitions and the lack of standard assessment tools, as well as of widely acceptable cut-offs to diagnose SCD, the content validity of our questionnaires (which are different than other studies) needs to be further assessed. Notably, there is a risk of selection bias in the ALBION study, as some participants were self-referred to the outpatient clinic. Except for this, by using PRSs based on GWASs to estimate genetic predisposition for Aβ_42_ and Tau, we were able to explore only the effect of the most common SNPs (and consequently variants), while other types of genetic variation and epigenetic influences, such as methylation [49] or metabolomics [50], could not be examined. Finally, CSF biomarkers were treated as categorical variables with the above-mentioned diagnostic cut-offs, which might be different to the cut-off points used in other studies. Additional studies performed in different ethnic groups and larger population-based groups of individuals are required to confirm our findings.

## 5. Conclusions

Although SCD has been reported in many studies as an early pre-dementia stage, it remains unknown whether AD-related pathology is, indeed, present in that early phase of the disease. Our study highlighted that changes in CSF Aβ_42_ increase the odds of prevalent SCD, while Tau (neither CSF nor total) does not appear to be related to SCD odds. Hence, SCD might be an intermediate stage between CN and MCI in which amyloidosis is present, while tauopathy is not. This is the first study to combine genetic predisposition for Aβ_42_ with actual CSF Aβ_42_ measurements, providing a more comprehensive approach to examining the association of Aβ_42_ with SCD, as well as new clues for the early identification of subjects in the preclinical AD stage. Further prospective research is needed to improve our understanding of the evolution of SCD over time. 

## Figures and Tables

**Table 1:** Subjective Cognitive Decline (SCD) assessment.

Questions Regarding Subjective Cognitive Complaints	
HELIAD	1. Do you have symptoms of memory loss? 2. Do you have difficulty in recalling recent events?
ALBION	1. Do you feel that your memory is worse than 5 years ago? 2. Do you feel that your memory is worse than peers?

**Table 2:** Descriptive statistics for HELIAD participants in groups according to presence of SCD.

	All	SCD ^1^ Group	Non-SCD	
	*n* = 742	*n* = 186	*n* = 556	*p*-Value
Age, years, mean ± SD ^2^	73.7 ± 5.2	73.6 ± 5.4	73.7 ± 5.1	0.819
Sex, female (%)	438 (59.0)	115 (61.8)	323 (58.1)	0.390
Education, years, mean ± SD	7.2 ± 4.4	8.5 ± 4.7	6.8 ± 4.3	**<0.001**
Neuropsychological score, mean ± SD	−0.17 ± 0.72	−0.24 ± 0.73	0.02 ± 0.69	**<0.001**
Depression, yes (%)	47 (6.3)	21 (11.3)	26 (4.7)	**0.030**
PRS ^3^ Aβ_42_, high (%)	371 (50.0)	106 (57.0)	265 (47.7)	**0.028**
PRS Tau, high (%)	371 (50.0)	89 (47.8)	282 (50.7)	0.498

^1^ Subjective Cognitive Decline, ^2^ Standard Deviation, ^3^ Polygenic Risk Score. Bold values indicate statistically significant differences between the two groups.

**Table 3:** Descriptive statistics for ALBION participants in groups according to presence of SCD.

	All	SCD ^1^ Group	Non-SCD	
	*n* = 107	*n* = 78	*n* = 29	*p*-Value
Age, years, mean ± SD ^2^	62.6 ± 9.2	62.3 ± 9.5	63.2 ± 9.3	0.644
Sex, female (%)	75 (70.1)	55 (70.5)	20 (69.0)	0.877
Education, years, mean ± SD	14.2 ± 3.6	13.8 ± 3.7	14.8 ± 3.3	0.238
Neuropsychological score, mean ± SD	0.266 ± 0.533	−0.003 ± 0.534	0.099 ± 0.577	0.391
Depression, yes (%)	28 (26.2)	24 (30.8)	4 (13.8)	0.076
CSF ^3^ Aβ_42_, abnormal (%)	48 (43.9)	40 (51.3)	8 (27.6)	**0.038**
CSF Tau, abnormal (%)	18 (16.8)	12 (15.4)	6 (20.7)	0.671
CSF P-Tau, abnormal (%)	14 (13.1)	11 (14.1)	3 (10.3)	0.595

^1^ Subjective Cognitive Decline, ^2^ Standard Deviation, ^3^ Cerebrospinal Fluid. Bold values indicate statistically significant differences between the two groups.

**Table 4:** Logistic regression results for factors affecting the odds of SCD in HELIAD and ALBION.

	HELIAD, OR ^1^ (95% CI ^2^)	ALBION, OR (95%CI)
Age, years	**1.027 (1.017, 1.037), *p* < 0.001**	1.012 (0.984, 1.040)
Sex (male as reference)	1.203 (0.858, 1.548)	1.195 (0.789, 1.601)
Education, years	1.047 (0.988, 1.106)	0.967 (0.871, 1.073)
Neuropsychological score	0.626 (0.118, 1.134)	0.667 (0.274, 1.060)
Depression (no as reference)	**1.133 (1.044, 1.222), *p* = 0.027**	1.464 (0.891, 2.037)
PRS ^3^ Aβ_42_ (low as reference)	**1.689 (1.487, 1.891), *p* = 0.035**	-
PRS Tau (low as reference)	1.144 (0.954, 1.334)	-
CSF ^4^ Aβ_42_ (normal as reference)	-	**2.583 (1.020, 4.047),** ***p* = 0.045**
CSF Tau (normal as reference)	-	1.521 (0.805, 2.236)
CSF P-Tau (normal as reference)	-	1.149 (0.407, 1.891)

^1^ Odds Ratio, ^2^ Confidence Intervals, ^3^ Polygenic Risk Score, ^4^ Cerebrospinal Fluid. Bold values indicate statistically significant differences between the two groups.

## Data Availability

The data that support the findings of this study are available from the study’s principal investigator, N.S., upon reasonable request.

## Supplementary Materials

The following supporting information can be downloaded at https://www.mdpi.com/article/10.3390/biomedicines12051053/s1. Table S1: Number of SNPs included at each PRS Aβ_42_ calculated at different GWAS *p*-value thresholds. AUC area together with *p*-value of each PRS derived from a logistic regression with SCD as outcome, adjusted for PC1 and PC2. Table S2: Number of SNPs included at each PRS Tau calculated at different GWAS *p*-value thresholds. AUC area together with *p*-value of each PRS derived from a logistic regression with SCD as outcome, adjusted for PC1 and PC2. References [33,51,52,53,54,55,56,57,58,59,60,61,62] are cited in the supplementary materials.
