# Supplementary material for: Cerebral Amyloidosis in Individuals with Subjective Cognitive Decline: From Genetic Predisposition to Actual Cerebrospinal Fluid Measurements
Source: Biomedicines. 2024 May 10;12(5):1053. doi: 10.3390/biomedicines12051053 (PMC11118196; doi:10.3390/biomedicines12051053)
Supplement: Supplementary file 1 [file biomedicines-12-01053-s001.zip › biomedicines-2974325-supplementary.pdf]

Supplementary Materials

Table S1: Number of SNPs included at each PRS Aβ_{42} calculated at different GWAS p-value thresholds. AUC area together with p-value of each PRS derived from a logistic regression with SCD as outcome, adjusted for PC1 and PC2.

PRS pT	Number of SNPs	AUC	p-value
PRS 5e-5	30	0.566	**0.040**
PRS 0.0001	57	0.581	**0.024**
PRS 0.001	546	0.547	0.112
PRS 0.01	3879	0.550	0.104
PRS 0.05	13957	0.535	0.186
PRS 0.1	23399	0.538	0.164
PRS 0.2	38349	0.527	0.214
PRS 0.3	49846	0.541	0.144
PRS 0.4	59148	0.542	0.140
PRS 0.5	66573	0.531	0.202

Table S2: Number of SNPs included at each PRS Tau calculated at different GWAS p-value thresholds. AUC area together with p-value of each PRS derived from a logistic regression with SCD as outcome, adjusted for PC1 and PC2.

PRS pT	Number of SNPs	AUC	p-value
PRS 5e-8	21	0.578	**0.028**
PRS 5e-5	92	0.569	**0.038**
PRS 0.0001	144	0.534	0.180
PRS 0.001	1012	0.560	**0.046**
PRS 0.01	6906	0.528	0.204
PRS 0.05	25193	0.529	0.201
PRS 0.1	41878	0.522	0.242
PRS 0.2	67150	0.543	0.128
PRS 0.3	86412	0.532	0.176
PRS 0.4	101834	0.535	0.182
PRS 0.5	114769	0.533	0.178

Genotyping and Imputation in HELIAD

Genome-wide genotyping was performed at Life & amp Brain facilities (Germany) using the Illumina Infinium Global Screening Array and calling was generated by the "centre national de recherche en génétique humaine" (Evry, France) using the data generated by the centers involved in genotyping (Life & amp Brain, CNRGH and Erasmus Medical Center).

Briefly, variants included in the removal marker list by Illumina were excluded and only variants for which the full-length probes aligned uniquely on GRCh38 genome without mismatches were kept. Variant intensity quality control was conducted for all autosomal variants, according to established thresholds, while sex-check was performed using chrX

variants [1]. Samples with missingness > 0.05, sex inconsistencies or with heterozygosity rate that deviated > ± 6 Standard Deviation (SD) from the mean, were excluded. To identify population outliers, we ran Principal Component Analysis, using as reference dataset the 1000 Genome population and the combined dataset was projected onto two dimensions, using flashPCA2 [2]. To control for cryptic relatedness, we excluded one individual from each pair of samples with a kinship coefficient more than 0.125 (cut-off for second-degree relatives), yielding a final sample size of 1251 unrelated individuals. We excluded variants with a missingness >0.05 in at least one genotyping center or having a differential missingness test $P<10^{-10}$. The Hardy-Weinberg equilibrium tests ($p<5e^{-8}$) were performed only in controls and for each genotyping center/country separately.

To improve the accuracy of imputation, we compared the frequencies of variants (Chi Square test) against two reference panels, the population of the Haplotype Reference Consortium r1.1 (HRC) [3] excluding samples from the 1000 Genomes and the Finnish and the non-Finnish population of Genome Aggregation Database v3 (gnomAD) [4]. Variants showing a x^2 >3,000 in both HRC and gnomAD or in one reference panel without being present in the other were excluded. Finally, GWASs were performed between controls across genotyping centers to assess frequency differences between genotyping centers, using SNPTEST [5], under an additive model and adjusting on associated Principal Components. Variants with a Likelihood Ratio Test of $p < 10^{-5}$ were excluded. Furthermore, we removed ambiguous variants with Minor Allele Frequency (MAF) > 0.4 and we kept only one copy of any duplicated variants, prioritizing the one with the lowest missingness. All qualified samples and variants were imputed on Michigan Imputation Server (v1.2.4) [6], using the TOPMed Freeze 5 reference panel. Phasing and imputation were performed using EAGLE v2.4 [7] and Minimac4 v4-1.0.2 software, respectively.

Polygenic Risk Score (PRS) Calculation

In the HELIAD genotype data and prior to PRS calculation, imputed SNPs dosages for a total of 5,611,082 SNPs, with MAF>0.05, call rate >95% and imputation quality score >0.4 were converted to best-guess genotypes (with probability >0.8). The PRSice software (http://prsice.info/) [8] was utilized to construct PRSs for each HELIAD participant applying the clumping and thresholding (C+T) method, following the approach originally described by the International Schizophrenia Consortium [9]. In particular, the risk score of each SNP is calculated by multiplying the risk allele number (0,1,2) with the corresponding effect size [the log of Odd Ratio (OR)] for the reference allele reported in the GWAS summary data [10]. Effect size is used as a weight of the risk that each SNP confers and the PRS for each individual is computed as the sum of log (OR)-weighted genotypes of all SNPs. To ensure that only independent markers are included in the computed PRS score, we first clumped SNPs of the HELIAD data for linkage disequilibrium (SNPs with r^2=0.1 in 250 kb-windows were removed). Markers within the major histocompatibility complex (MHC) LD region on chromosome 6 (chr6:27-33Mb, hg19) were also excluded from PRS computation process due to the high polymorphic nature of this region. It is well known that APOE genotype is a critical component of AD and predicts amyloid deposition [11]. When PRS is calculated including APOE region its effect appeared to be driven by APOE and may not be more predictive than APOE alone. To capture the effects of common non-APOE variants and similar with previous studies [12,13],

PRS was calculated excluding *APOE* region, defined as 1MB up and downstream of the gene (chr19: 44,409,039-46,412,650) and APOE genotype was added in the model as covariate.

References

1. Grove ML, Yu B, Cochran BJ, et al. Best Practices and Joint Calling of the HumanExome BeadChip: The CHARGE Consortium. *PLOS ONE*. 2013;8(7):e68095. doi:10.1371/journal.pone.0068095
2. Abraham G, Qiu Y, Inouye M. FlashPCA2: principal component analysis of Biobank-scale genotype datasets. *Bioinformatics*. 2017;33(17):2776-2778. doi:10.1093/bioinformatics/btx299
3. McCarthy S, Das S, Kretzschmar W, et al. A reference panel of 64,976 haplotypes for genotype imputation. *Nat Genet*. 2016;48(10):1279-1283. doi:10.1038/ng.3643
4. Karczewski KJ, Francioli LC, Tiao G, et al. The mutational constraint spectrum quantified from variation in 141,456 humans. *Nature*. 2020;581(7809):434-443. doi:10.1038/s41586-020-2308-7
5. Marchini J, Howie B, Myers S, McVean G, Donnelly P. A new multipoint method for genome-wide association studies by imputation of genotypes. *Nat Genet*. 2007;39(7):906-913. doi:10.1038/ng2088
6. Das S, Forer L, Schönherr S, et al. Next-generation genotype imputation service and methods. *Nat Genet*. 2016;48(10):1284-1287. doi:10.1038/ng.3656
7. Loh PR, Danecek P, Palamara PF, et al. Reference-based phasing using the Haplotype Reference Consortium panel. *Nat Genet*. 2016;48(11):1443-1448. doi:10.1038/ng.3679
8. Choi SW, O'Reilly PF. PRSice-2: Polygenic Risk Score software for biobank-scale data. *Gigascience*. 2019;8(7):giz082. doi:10.1093/gigascience/giz082
9. International Schizophrenia Consortium, Purcell SM, Wray NR, et al. Common polygenic variation contributes to risk of schizophrenia and bipolar disorder. *Nature*. 2009;460(7256):748-752. doi:10.1038/nature08185
10. Deming Y, Li Z, Kapoor M, et al. Genome-wide association study identifies four novel loci associated with Alzheimer's endophenotypes and disease modifiers. *Acta Neuropathol*. 2017;133(5):839-856. doi:10.1007/s00401-017-1685-y
11. Morris JC, Roe CM, Xiong C, et al. APOE predicts amyloid-beta but not tau Alzheimer pathology in cognitively normal aging. *Annals of Neurology*. 2010;67(1):122-131. doi:10.1002/ana.21843
12. Moore A, Filshtein T, Dumitrescu L, et al. A/T/N polygenic risk score for cognitive decline in old ag. 2019. doi:10.1101/838847
13. Kennedy G, Moore AM, Mahoney ER, et al. CSF polygenic risk AD biomarkers predict brain amyloid and free recall. *Alzheimer's & Dementia*. 2021;17(S4):e056252. doi:10.1002/alz.056252

Disclaimer/Publisher's Note: The statements, opinions and data contained in all publications are solely those of the individual author(s) and contributor(s) and not of MDPI and/or the editor(s). MDPI and/or the editor(s) disclaim responsibility for any injury to people or property resulting from any ideas, methods, instructions or products referred to in the content.
